# Physician Compliance With a Computerized Clinical Decision Support System for Anemia Management of Patients With End-stage Kidney Disease on Hemodialysis: Retrospective Electronic Health Record Observational Study

**DOI:** 10.2196/44373

**Published:** 2023-05-03

**Authors:** Ju-Yeh Yang, Kai-Hsiang Shu, Yu-Sen Peng, Shih-Ping Hsu, Yen-Ling Chiu, Mei-Fen Pai, Hon-Yen Wu, Wan-Chuan Tsai, Kuei-Ting Tung, Raymond N Kuo

**Affiliations:** 1 Institute of Health Policy and Management College of Public Health National Taiwan University Taipei Taiwan; 2 Division of Nephrology Department of Internal Medicine Far Eastern Memorial Hospital New Taipei City Taiwan; 3 Center for General Education Lee-Ming Institute of Technology New Taipei City Taiwan; 4 Graduate Institute of Medicine Yuan Ze University Taoyuan Taiwan; 5 Graduate Program in Biomedical Informatics Yuan Ze University Taoyuan Taiwan

**Keywords:** clinical decision support system, erythropoietin-stimulating agent, end-stage kidney disease, hemodialysis, physician compliance, kidney disease, clinical decision support, electronic health records, decision support, anemia management, patient outcome

## Abstract

**Background:**

Previous studies on clinical decision support systems (CDSSs) for the management of renal anemia in patients with end-stage kidney disease undergoing hemodialysis have previously focused solely on the effects of the CDSS. However, the role of physician compliance in the efficacy of the CDSS remains ill-defined.

**Objective:**

We aimed to investigate whether physician compliance was an intermediate variable between the CDSS and the management outcomes of renal anemia.

**Methods:**

We extracted the electronic health records of patients with end-stage kidney disease on hemodialysis at the Far Eastern Memorial Hospital Hemodialysis Center (FEMHHC) from 2016 to 2020. FEMHHC implemented a rule-based CDSS for the management of renal anemia in 2019. We compared the clinical outcomes of renal anemia between the pre- and post-CDSS periods using random intercept models. Hemoglobin levels of 10 to 12 g/dL were defined as the on-target range. Physician compliance was defined as the concordance of adjustments of the erythropoietin-stimulating agent (ESA) between the CDSS recommendations and the actual physician prescriptions.

**Results:**

We included 717 eligible patients on hemodialysis (mean age 62.9, SD 11.6 years; male n=430, 59.9%) with a total of 36,091 hemoglobin measurements (average hemoglobin and on-target rate were 11.1, SD 1.4, g/dL and 59.9%, respectively). The on-target rate decreased from 61.3% (pre-CDSS) to 56.2% (post-CDSS) owing to a high hemoglobin percentage of >12 g/dL (pre: 21.5%; post: 29%). The failure rate (hemoglobin <10 g/dL) decreased from 17.2% (pre-CDSS) to 14.8% (post-CDSS). The average weekly ESA use of 5848 (SD 4211) units per week did not differ between phases. The overall concordance between CDSS recommendations and physician prescriptions was 62.3%. The CDSS concordance increased from 56.2% to 78.6%. In the adjusted random intercept model, the post-CDSS phase showed increased hemoglobin by 0.17 (95% CI 0.14-0.21) g/dL, weekly ESA by 264 (95% CI 158-371) units per week, and 3.4-fold (95% CI 3.1-3.6) increased concordance rate. However, the on-target rate (29%; odds ratio 0.71, 95% CI 0.66-0.75) and failure rate (16%; odds ratio 0.84, 95% CI 0.76-0.92) were reduced. After additional adjustments for concordance in the full models, increased hemoglobin and decreased on-target rate tended toward attenuation (from 0.17 to 0.13 g/dL and 0.71 to 0.73 g/dL, respectively). Increased ESA and decreased failure rate were completely mediated by physician compliance (from 264 to 50 units and 0.84 to 0.97, respectively).

**Conclusions:**

Our results confirmed that physician compliance was a complete intermediate factor accounting for the efficacy of the CDSS. The CDSS reduced failure rates of anemia management through physician compliance. Our study highlights the importance of optimizing physician compliance in the design and implementation of CDSSs to improve patient outcomes.

## Introduction

Anemia is one of the most important complications of kidney failure and is associated with significant morbidity, mortality, and cost. Modern therapeutics such as recombinant erythropoietin-stimulating agent (ESA) and hypoxia inducible factor propylhydroxylase inhibitor [1] provide pharmacologic treatment for anemia in patients with end-stage kidney disease (ESKD). ESA treatment has been proven to improve quality of life [2] and reduce hospitalization [3] and mortality [4]. However, optimal management of anemia remains challenging. Current clinical guidelines recommend an optimal hemoglobin (Hb) range of 10-11 g/dL [5-7]. Underdosing of ESA fails to maintain optimal Hb range, and overdosing of ESA increases health care costs and is associated with increased mortality [8,9].

ESA responsiveness in patients with ESKD is considered “complex, non-linear, and dynamic” [10] with high interpersonal variability. Several parameters are associated with the responsiveness of ESA, including iron status [11], age, sex, comorbidity, BMI, nutritional status, chronic inflammation, heart failure, etc [10]. The complex and nonlinear relationship between anemia-mitigating therapeutics and treatment responses has prompted researchers to develop several clinical decision support system (CDSS) programs to address this clinically challenging problem. Typically, CDSSs are developed based on expert-driven decision rules or data-driven decision algorithms to improve health care implementation or patient outcomes. Nevertheless, the most important factor that hinders the successful implementation of the CDSS is physician compliance. Previous CDSS studies have focused mainly on comparing the treatment effects of CDSS [12-15]. Brier et al [16] conducted a randomized controlled trial of a CDSS and usual care. Miskulin et al [17] conducted a cluster analysis with a control group. Four other studies used an observational study design to compare the results before and after CDSS [18-21]. These prior studies revealed the inconsistent effects of CDSSs, including decreasing Hb variability, increasing compliance with Hb targets, reducing ESA dose, and reducing workload [14,16,17,20,22,23]. However, we could not infer the impact of physicians’ compliance on CDSS efficacy based on their findings.

In theory, the CDSS is designed to collect and integrate clinical information and then generate actionable recommendations for clinical decision-making to support frontline health care providers. However, real-world evidence suggests that the CDSS has limited applications for change in the behavior of frontline health care providers. A systematic analysis investigating 166 randomized controlled trials revealed that only 52%-64% and 15%-31% of studies indicated that CDSSs either changed the health care processes or actually improved patient prognosis [24], respectively. Adequate evidence confirms that compliance with CDSSs is a critical factor in determining whether CDSSs can change health care providers’ behavior [25-29]. However, none of the aforementioned studies investigated the important role between physician compliance with CDSSs and CDSS treatment effects.

To address this knowledge gap, we conducted this study to evaluate the impact of physician compliance with CDSSs for anemia management in patients with ESKD undergoing hemodialysis.

## Methods

### Data and Population

**Figure 1:**
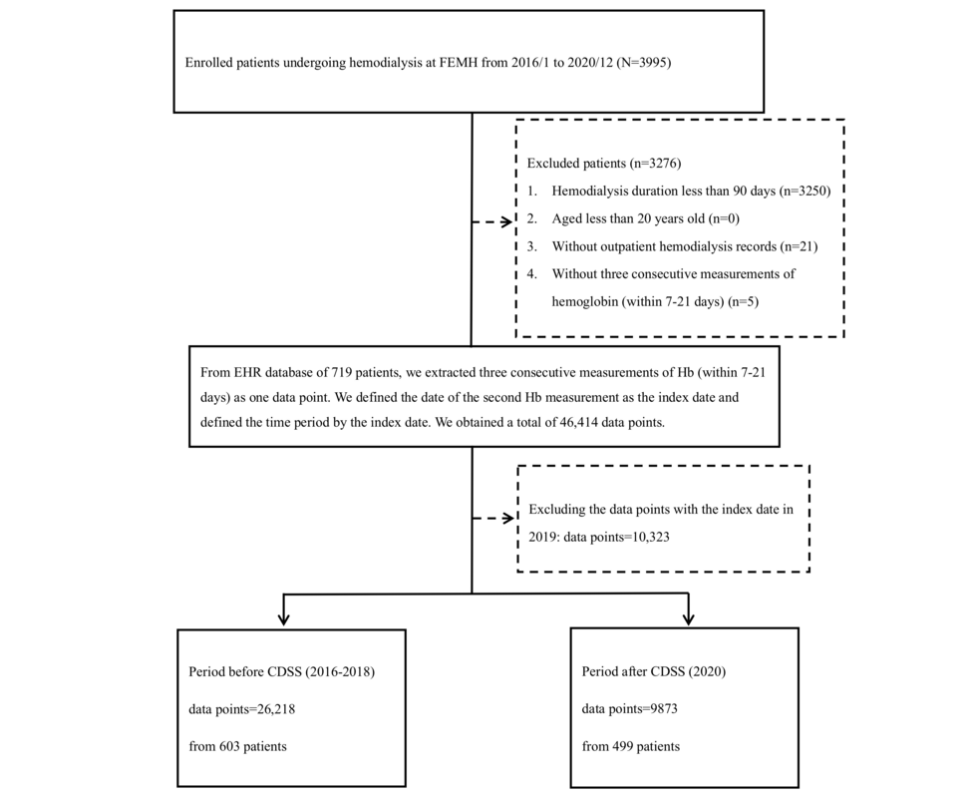
Flowchart of patients and data points. CDSS: clinical decision support system; EHR: electronic health record; FEMH: Far Eastern Memorial Hospital; Hb: hemoglobin.

We extracted the electronic health records of patients with ESKD undergoing hemodialysis at the Far Eastern Memorial Hospital (FEMH) medical center from 2016 to 2020. Patients with ESKD were defined as those on stable hemodialysis for >3 months. Standard laboratory tests included Hb measurements twice per month and biochemical measurements once per month. We included only adult patients (age >20 years) who had outpatient dialysis records and at least three eligible Hb measurements. For each eligible “index-Hb,” we extracted a “pre-Hb” and “post-Hb” record defined as Hb measured 2 weeks before and 2 weeks after the index date, respectively. Three sequential eligible Hb measurements (pre-Hb, index Hb, and post-Hb) constituted a complete observational data point for analysis. A flowchart of cohort establishment is shown in Figure 1.

### CDSS for ESA Prescription

The FEMH Hemodialysis Center (FEMHHC) has been implementing electronic health records since 2016. In 2019, the FEMHHC adopted the computerized CDSS for the prescriptions of ESA and iron supplements (Supplements 1 and 2 in Multimedia Appendix 1).

The CDSS implemented in FEMHHC in 2019 is a ruled-based CDSS developed and modified by nephrology experts. Specifically, the rule-based CDSS algorithms would input the prior Hb levels, latest Hb level, changes in Hb levels, and prior prescriptions in the past 2 weeks; then the CDSS would calculate the change of Hb and iron profile under the ESA dose. Finally, the CDSS would output recommended doses of ESA and iron supplement for physicians to consider. The physicians would still have to make the final prescriptions on the doses of ESA and iron supplement. At the physician’s discretion, the physician could accept the CDSS-recommended doses or prescribe different doses. There was neither interruption in the workflow nor administrative intervention (encouragement or punishment) for physician compliance with the CDSS.

### Study Outcomes

The CDSS program was implemented at FEMH in 2019. Therefore, we divided the data into three chronological phases: the pre-CDSS phase (from 2016 to 2018), the CDSS implementation phase (2019), and the post-CDSS phase (2020). We compared the indicators (absolute Hb level, on-target rate [defined as the percentage of Hb levels between 10 and 12 g/dL], failure rate [defined as Hb<10 g/dL]) and anemia control behaviors (weekly ESA dosage, ESA prescription change rate, and CDSS concordance rate) in the pre- and post-CDSS phases. Concordance with the CDSS was defined as <50% for the difference between ESA suggested dose (according to the CDSS) and the prescribed physician ESA dose in the same direction (addition or reduction in doses). We used the concordance rate as an indicator of physician compliance with the CDSS.

### Statistical Section and Missing Data Handling Method

Considering the dependence between repeated measurements within individuals, we used random intercept mixed models to examine differences in the distributions of all variables during the pre- and post-CDSS phases. All covariates were included in the full model to adjust for confounding factors that might interfere with the primary outcomes. The covariates of interest included weight; weight gain; heparin use; types of vascular access; dialysis frequency; and serum levels of albumin, calcium, phosphate, potassium, parathyroid hormone, ferritin, and iron saturation.

For missing Hb data, we would discard this observation because Hb is the key outcome variable in this study. For missing laboratory data, we would use the last laboratory data within the previous 3 months.

We further added CDSS concordances as an intermediate variable in the full model to assess the role of CDSS concordance in the causal relationship between CDSS implantation and parameters of anemia controls. Observed power was calculated according to Cohen [30,31].

We applied an interaction term in the full model to determine whether age and weight modified the changes in anemia control in the pre-CDSS and post-CDSS phases. All analyses were performed using the SAS software (version 9.4; SAS Institute Inc).

### Ethical Considerations

The FEMH Research Ethics Committee waived the requirement for informed consent in this retrospective observational study on electronic health records (FEMH No. 109178-E). All methods were carried out in accordance with the Declaration of Helsinki and with relevant guidelines and regulations. The study data were deidentified, and there was no compensation for study subjects.

## Results

We included 717 eligible hemodialysis patients with 36,091 Hb measurements during the study period (Table 1). Each patient contributed 19.5 measurements of Hb per year, their mean age was 62.9 (SD 11.6) years, and the number of male patients was 430 (59%). More than half (n=418, 58.2%) of the patients were diabetic, and 187 (26.1%) had chronic hepatitis B or C. Most patients (n=603, 84%) received hemodialysis via an arteriovenous fistula. Ferritin levels were higher in the post-CDSS than pre-CDSS (455 vs 342 ng/dL) stage. Other biochemical test results were not significantly different in the pre-CDSS and post-CDSS phases.

The average Hb was 11.1 (SD 1.4) g/dL and the on-target rate (Hb 10-12 g/dL) was 59.9%. The on-target rate decreased post CDSS (pre vs post: 61.3% vs 56.2%) owing to a higher percentage of Hb >12 g/dL (pre vs post: 21.5% vs 29%). The failure rate (Hb <10 g/dL) decreased post CDSS (pre vs post: 17.2% vs 14.8%). The average weekly ESA use of 5848 (SD 4211) units per week did not differ between the two phases.

When new Hb measurement data were available, ESA prescriptions were adjusted in 7198 of 36,091 (19.9%) observations. In the post-CDSS phase, the prescription adjustment rate increased nearly 2-fold (4072/26,218, 15.5% vs 3126/9873, 31.7%). The overall concordance between the CDSS recommendations and physician prescriptions was 22,492 of 36,091 (62.3%). Further, we included the laboratory and prescription data in the pre-CDSS phase into the model to derive CDSS recommendations for the pre-CDSS phase prescriptions. We then compared the actual prescriptions and CDSS-recommended prescriptions in this pre-CDSS phase to calculate the concordance rate. The CDSS concordance in the pre- and post-CDSS phases increased from 14,734 of 26,218 (56.2%) to 7758 of 9873 (78.6%).

After controlling for all the variables in the random intercept model (Table 2), the comparison of results of the post-CDSS phase with those of the pre-CDSS phase showed increased Hb by 0.17 (95% CI 0.14-0.21) g/dL, weekly ESA use increased by 264 (95% CI 158-371) units per week, the on-target rate reduced by 29% (odds ratio [OR] 0.71, 95% CI 0.66-0.75), failure rate reduced by 16% (OR 0.84, 95% CI 0.76-0.92), and the concordance rate increased 3.4-fold (95% CI 3.1-3.6).

Concordance with CDSS suggestions is an important mediator between introducing CDSS and the performance of all indicators of anemia management (Table 3). After additional adjustments for concordance in the full models, the increases in Hb and the decrease in the on-target rate tended to be attenuated (Hb changed from 0.17 to 0.13 g/dL, whereas the OR of the on-target rate reduced from 0.71 to 0.73). The increases in ESA and decreases in failure rate were completely mediated by the CDSS concordance (changes in ESA dose from 264 to 50 units; OR of failure rates from 0.84 to 0.97).

**Table 1:** Characteristics of study participants.

		Total (2016-2018, 2020; N=717)	By period	
			Pre-CDSS^a^ phase (2016-2018; n=603)	Post-CDSS phase (2020; n=499)
Data count, n		36,091	26,218	9873
Measurements per patient-year, mean (SD)		19.5 (6.5)	19.4 (6.8)	19.8 (5.8)
Age (years), mean (SD)		62.9 (11.6)	62.7 (11.5)	63.4 (11.8)
Male, n (%)		21,317 (59.1)	15,325 (58.5)	5992 (60.7)
Diabetes mellitus, n (%)		20,996 (58.2)	15,039 (57.4)	5957 (60.3)
Hepatitis B or C, n (%)		9430 (26.1)	7026 (26.8)	2404 (24.4)
Weight (kg), mean (SD)		63.8 (13.4)	63.3 (13.4)	65.4 (13.4)
Weight gain (kg), mean (SD)		2.1 (1.3)	2.2 (1.3)	2.0 (1.4)
Heparin (U/session), median (IQR)		1000 (0-1750)	1000 (0-1750)	1250 (0-2000)
Vascular access (AVF^b^/AVG^c^/catheter), %		84.1/12.7/7.4	84/11.8/9.9	84.2/15.2/0.9
Dialysis twice per week, n (%)		1043 (2.9)	732 (2.8)	311 (3.1)
Albumin (g/dL), mean (SD)		3.9 (0.4)	3.9 (0.4)	4.0 (0.4)
Ca (mg/dL), mean (SD)		9.2 (0.7)	9.2 (0.7)	9.1 (0.8)
P (mg/dL), mean (SD)		4.8 (1.4)	4.8 (1.4)	5.0 (1.4)
K (mEq/L), mean (SD)		4.4 (0.7)	4.4 (0.7)	4.4 (0.7)
PTH^d^ (pg/mL), median (IQR)		207.7 (84.8-420.0)	194.6 (80.7-397.6)	242.5 (100.5-480.8)
Ferritin (ng/dL), mean (SD)		373.1 (339.2)	342 (305.1)	455.5 (405)
Iron/TIBC^e^ (%), mean (SD)		27.6 (11.7)	27 (11.3)	29.1 (12.5)
**Hemoglobin**				
	Hemoglobin level, mean (SD)	11.1 (1.4)	11.1 (1.4)	11.3 (1.4)
	On-target rate (10≤hemoglobin≤12 g/dL)	59.9	61.3	56.2
	Hemoglobin>12 g/dL (%)	23.6	21.5	29.0
	Failure rate (hemoglobin<10 g/dL)	16.5	17.2	14.8
**ESA^f^**				
	ESA order (U/week), mean (SD)	5848 (4211)	5828 (4054)	5839 (4613)
	ESA order changed, n (%)	7198 (19.9)	4072 (15.5)	3126 (31.7)
	CDSS concordance, n (%)	22,492 (62.3)	14,734 (56.2)	7758 (78.6)

^a^CDSS: clinical decision support system.

^b^AVF: arteriovenous fistula.

^c^AVG: arteriovenous graft.

^d^PTH: parathyroid hormone.

^e^TIBC: total iron-binding capacity

^f^ESA: erythropoietin-stimulating agent.

**Table 2:** Estimates of the effect of the clinical decision support system in random intercept models.

		Estimates (95% CI)	Observed power
**Changes in hemoglobin (g/dL)**			
	Univariate	0.18 (0.15 to 0.21)	0.99
	Multivariate^a^	0.17 (0.14 to 0.21)	0.99
**Changes in erythropoietin-stimulating agent dosage (unit/week)**			
	Univariate	–269 (–363 to –175)	0.99
	Multivariate^a^	264 (158 to 371)	0.99
**Odds ratio of on-target rate**			
	Univariate	0.81 (0.76 to 0.86)	0.99
	Multivariate^a^	0.71 (0.66 to 0.75)	0.99
**Odds ratio of failure rate**			
	Univariate	0.82 (0.75 to 0.89)	0.99
	Multivariate^a^	0.84 (0.76 to 0.92)	0.92
**Odds ratio of concordance rate**			
	Univariate	3.1 (2.9 to 3.3)	0.99
	Multivariate^a^	3.4 (3.1 to 3.6)	0.99

^a^Adjusted for age, sex, diabetes mellitus, viral hepatitis, albumin, calcium, phosphate, potassium, parathyroid hormone, ferritin, iron saturation, catheter use, weight, weight gain, dialysis frequency, and heparin use.

**Table 3:** Intermediate effect of concordance (physician compliance with the clinical decision support system).

		Estimates (95% CI)	*P* value comparing model 1 vs model 2	Observed power
**Changes in hemoglobin (g/dL)**			<.001	0.99
	Model 1^a^	0.17 (0.14 to 0.21)		
	Model 2^b^	0.13 (0.10 to 0.17)		
**Changes in erythropoietin-stimulating agent dosage per week (unit/week)**			<.001	0.99
	Model 1^a^	264 (158 to 371)		
	Model 2^b^	50 (–57 to 157)		
**Odds ratio of on-target rate**			<.001	0.99
	Model 1^a^	0.71 (0.66 to 0.75)		
	Model 2^b^	0.73 (0.68 to 0.77)		
**Odds ratio of failure rate**			<.001	0.99
	Model 1^a^	0.84 (0.76 to 0.92)		
	Model2^b^	0.97 (0.88 to 1.07)		

^a^Model 1: adjusted for age, sex, diabetes mellitus, viral hepatitis, albumin, calcium, phosphate, potassium, parathyroid hormone, ferritin, iron saturation, catheter use, weight, weight gain, dialysis frequency, and heparin use.

^b^Model 2: model 1 plus concordance.

## Discussion

To the best of our knowledge, our CDSS study has the largest number of observation data points (n=36,091 Hb measurements) from 717 patients with ESKD on hemodialysis in a single medical center over a period of 4 years. Our study had three unique features: (1) good baseline anemia management status with <20% of patients having Hb <10 g/dL, (2) low baseline total ESA use with a mean ESA dose of <6000 IU per week, and (3) we used individual-level data and adjusted for interobservation dependence of multiple measurements using mixed models. Despite the limited room for improvement in anemia management owing to these features, our study showed that the CDSS still achieved clinical efficacy in reducing the failure rate. We demonstrated that concordance with the CDSS completely mediated its efficacy.

Although several studies on CDSSs for anemia management in patients with ESKD have been published, most have focused on the comparison between the effects of different CDSSs [12-15]. There is limited data on the verification of interventional efficacy of CDSSs in daily practice in hemodialysis units.

To study the efficacy of iron supplementation on anemia management, Richardson et al [21] developed a CDSS to calculate a recommended ESA dose for nurses to administer. After 24 months of observation, the 228 patients with ESKD on hemodialysis had similar Hb levels after CDSS implementation (compared with baseline data 3 months before the CDSS) and significantly reduced median ESA use (136 IU/kg/week to 72 IU/kg/week, *P*<.001). These study results suggest that the CDSS improved anemia management. However, the CDSS was directly used by nurses, and therefore, we could not investigate the impact of physician compliance on the efficacy of the CDSS.

Gaweda et al [19] used an artificial neural network to develop a model predictive control (MPC)–based CDSS. They observed the efficacy of the MPC-based CDSS in 9 patients, and the results showed that the on-target rate (Hb 10-12 g/dL) had an increasing trend and that ESA dosage had a decreasing trend, but both parameters failed to reach statistical significance. Brier et al [16] later conducted a randomized controlled trial to investigate the efficacy of the MPC-based CDSS. Of 60 patients with ESKD on hemodialysis (30 patients on the MPC-based CDSS, the remaining 30 patients on standard of care anemia management protocol), the MPC group had lower Hb variability, but the on-target rate was comparable between the two groups. The MPC-based CDSS group had a higher total ESA dose than the standard of care group (129,300 IU vs 97,600 IU). This study is the only randomized controlled study, but the physicians always received a recommended dose, either from the CDSS or from a nurse practitioner. We therefore could not evaluate the effect of physician compliance or acceptance on the efficacy of the CDSS.

Miskulin et al [17] compared the efficacy of a CDSS (n=1118 hemodialysis patients) with the efficacy of traditional physician prescriptions (n=7823 hemodialysis patients). During 6 months of observation, no difference was observed in the on-target rate of Hb and Hb variability. The self-administered questionnaire showed that the time spent by nurse managers on anemia management was less in the CDSS group (3 h/month vs 6.5 h/month; *P*<.001). This study did not investigate physician compliance with or acceptance of the CDSS.

Since 2008, Mayo Clinic adopted the Mayo Clinic Anemia Management System (MCAMS) in its own hemodialysis units [20]. They conducted an observational study including around 300 patients per month at 8 hemodialysis units. From the pre-MCAMS (2007) to post-MCAMS (2010) period, the Hb on-target rate increased from 69% to 83% (*P*<.001) and the monthly ESA dose decreased from 304 μg/month to 173 μg/month (*P*<.001). However, the percentage of patients with Hb <10 g/dL increased. This study used aggregated population-level data and did not evaluate individual variances over time or physician compliance with the CDSS.

Barbieri et al [18] conducted a retrospective observational study on the CDSS of the anemia control model in 752 patients with ESKD on hemodialysis in 3 countries. The total observation data points were 9501 measurements 1 year before CDSS implementation (control group) and 1 year after CDSS implementation (intervention group). The results showed that the Hb on-target rate increased (70.6% vs 76.6%; *P*<.001) and the total ESA dose decreased (0.63 vs 0.46 μg/kg/month; *P*<.001). In the subgroup analysis, the compliant subgroup (more than two-thirds accepting the CDSS recommendations) had a larger magnitude of improvement in anemia management, which confirmed the importance of CDSS compliance. Nevertheless, this study did not consider the cluster effect between different hemodialysis centers and interobservational dependence in the same patient.

Our results filled the knowledge gap in the role of physician compliance in the efficacy of CDSSs. Our data clearly confirmed that the CDSS indeed altered the prescription behavior of physicians. The odds of concordance tripled in the post-CDSS phase compared to that in the pre-CDSS phase. The CDSS reduced the failure rate of anemia management from 17.2% to 14.8%. After controlling for concordance with the CDSS, the significant difference in the failure rate between the pre- and post-CDSS phases disappeared, which indicated that the physicians’ compliance with the CDSS was a complete intermediate factor. Our results clearly showed that physician compliance impacted the efficacy of the CDSS.

Systematic reviews about the effects of CDSSs on practitioner performance and patient outcomes showed that around two-thirds of studies found a positive impact of CDSSs on practitioner performance, but CDSSs failed to show a positive impact on patient outcomes [32,33]. As artificial intelligence has been strengthening the robustness of CDSSs, a recent systematic review summarized that up to 61% of CDSS studies were associated with positive patient medical outcomes, while still only 66% of studies had a positive practitioner performance [34]. Such systematic reviews strongly suggested that CDSSs may have a distinct impact on practitioner performance and patient outcomes. Although CDSSs are designed to provide “decision support” for practitioners, implementation of CDSSs does not necessarily lead to improved practitioner performance or patient outcomes. While the optimized performance of CDSSs has been a common goal in developing CDSSs (eg, accuracy, sensitivity, specificity, area under the curve in diagnosis), we believe that it is time to emphasize and clarify the true impact of CDSSs on practitioner performance and patient outcomes in clinical settings.

Future research in CDSSs should target not only optimizing the performance but also improving physician compliance with CDSSs. Our study clearly showed that physician compliance was the key intermediary factor for the efficacy of CDSSs. Future studies might investigate the underlying factors influencing physician willingness to use the CDSS and physician compliance with the recommendations made by the CDSS. Some potential factors might deserve exploration, such as intrusion of daily practice, incentives for physicians, awareness, alert fatigue, and psychological senses of threat.

This study has some limitations. First, this was a retrospective study, and we did not record prospectively on each occasion whether the physician agreed or disagreed with the CDSS recommendation. Alternatively, we compared the actual physician prescriptions with the CDSS recommendations in a retrospective fashion. We assumed that the physician accepted the CDSS recommendation if the actual physician prescriptions were similar to the CDSS recommendations. This might lead to potential bias or imprecision, as we used the concordance rate as a proxy for physician compliance, which may overestimate the physician compliance. Nevertheless, in previously published studies, such concordance between physician prescriptions and CDSS recommendations has been adopted as an indicator of CDSS efficacy [35-37]. A direct and interruptive confirmation page in the CDSS system might be considered in future prospective studies. Second, this study used the calendar year as an instrumental variable to indicate whether the CDSS intervention occurred or not; however, the different time data points might involve historical bias, and it is difficult to make causal inference between changing indicators and the CDSS. Therefore, we adopted the concordance of the CDSS as an intermediate variable, and our data indirectly supported the concordance of the CDSS as the mechanism between the CDSS and anemia improvement. This finding also emphasizes the importance of physician compliance in evaluating the efficacy of the CDSS. Third, there were still approximately 20% of occasions in which physician prescriptions were not in concordance with CDSS recommendations. This implies that in 20% of cases, the physician’s behavior was not altered by the CDSS, which probably weakened the clinical efficacy of the CDSS. The discordance between the CDSS and physician prescriptions deserves further investigation to maximize the efficacy of the CDSS.

Our results confirmed that physician compliance was a complete intermediate factor accounting for the efficacy of the CDSS. The CDSS reduced failure rates of anemia management through physician compliance. Our study highlighted the importance of optimizing physician compliance in the design and implementation of CDSSs to improve patient outcomes. Further studies are warranted to address the factors influencing physician compliance with CDSSs to improve patient outcomes.

## Appendix

Multimedia Appendix 1 Dose recommendation algorithms for erythropoietin-stimulating agent and iron.

## Abbreviations

CDSS: clinical decision support system
ESA: erythropoietin-stimulating agent
ESKD: end-stage kidney disease
FEMH: Far Eastern Memorial Hospital
FEMHHC: Far Eastern Memorial Hospital Hemodialysis Center
Hb: hemoglobin
MCAMS: Mayo Clinic Anemia Management System
MPC: model predictive control
OR: odds ratio

## Data Availability

The data sets used or analyzed during this study are available from the corresponding author upon reasonable request.
