# Supplementary material for: Physician Compliance With a Computerized Clinical Decision Support System for Anemia Management of Patients With End-stage Kidney Disease on Hemodialysis: Retrospective Electronic Health Record Observational Study
Source: JMIR Form Res. 2023 May 3;7:e44373. doi: 10.2196/44373 (PMC10193219; doi:10.2196/44373)
Supplement: Multimedia Appendix 1 [file formative_v7i1e44373_app1.doc]

**Multimedia Appendix 1.** Dose recommendation algorithms for erythropoietin-stimulating agent and iron.

Supplement 1. ESA dose recommendation algorithms in the computerized decision support system (CDSS).

| Hb | previous ESA | σHb | σHb | ESA level (change) |
| --- | --- | --- | --- | --- |
| Hb ≥ 14 | ≠ Level 0 | ≥ 0 | σHb ≥ -1 | -3 levels than last prescription |
|  |  | 0σHb-1 | -1 > σHb ≥ -2 | -2 levels than last prescription |
|  |  | -1σHb-2 | -2 > σHb ≥ -3 | -1 levels than last prescription |
|  |  | <-2 | σHb < -3 | No change from last prescription |
|  | Level 0 |  |  | Level 0 |
| 14 > Hb ≥ 13 | ≠ Level 0 | ≥ 1 | σHb ≥ 0 | -3 levels than last prescription |
|  |  | 0σHb1 | 0 > σHb ≥ -1 | -2 levels than last prescription |
|  |  | -1σHb0 | -1 > σHb ≥ -2 | -1 levels than last prescription |
|  |  | <-1 | σHb <-2 | No change from last prescription |
|  | Level 0 |  |  | Level 0 |
| 13 > Hb ≥ 12 | ≠ Level 0 | ≥ 2 | σHb ≥ 1 | -3 levels than last prescription |
|  |  | 1σHb2 | 1 > σHb ≥ 0 | -2 levels than last prescription |
|  |  | 0σHb1 | 0 > σHb ≥ -1 | -1 levels than last prescription |
|  |  | <1 | σHb <-1 | No change from last prescription |
|  | Level 0 |  |  | Level 0 |
| 12 > Hb ≥ 11 | ≠ Level 0 | ≥ 2 | σHb >2 | -2 levels than last prescription |
|  |  | 1σHb2 | 2 > σHb ≥ 1 | -1 levels than last prescription |
|  |  | -1σHb1 | 1 > σHb ≥ -1 | No change from last prescription |
|  |  | < -1 | σHb <-1 | +1 levels than last prescription |
|  | Level 0 | ≥ -1 | σHb ≥ 0 | Level 0 |
|  |  | <-1 | 0 > σHb ≥ -1 | Level 1 |
|  |  |  | σHb < -1 | Level 2 |
|  |  | N/A | **n/a** | Level 0 |
| 11 > Hb ≥ 10 | ≠ Level 0 | ≥ 1 | σHb ≥ 2 | -1 levels than last prescription |
|  |  | -1σHb1 | 2 > σHb ≥ 0 | No change from last prescription |
|  |  | < -1 | 0 > σHb ≥ -1 | +1 level than last prescription |
|  |  |  | -1 > σHb ≥ -2 | +2 levels than last prescription |
|  |  |  | σHb < -2 | +3 levels than last prescription |
|  | Level 0 | ≥ 0 | σHb ≥ 1 | Level 2 |
|  |  |  | 1 > σHb ≥ 0 | Level 3 |
|  |  | <0 | 0 > σHb ≥ -1 | Level 4 |
|  |  |  | -1 > σHb ≥ -2 | Level 5 |
|  |  |  | σHb < -2 | Level 6 |
|  |  | N/A | **n/a** | Level 4 |
| 10 > Hb ≥ 9 | ≠ Level 0 | > 0 | σHb > 1 | No change from last prescription |
|  |  |  | 1 > σHb ≥ 0 | +1 levels than last prescription |
|  |  | <-1 | 0> σHb ≥ -1 | +2 levels than last prescription |
|  |  | <-2 | σHb < -1 | +3 levels than last prescription |
|  | level0 | ≥ 0 | σHb ≥ 0 | Level 7 |
|  |  | <0 | σHb < 0 | Level 8 |
|  |  | N/A | **n/a** | Level 7 |
| Hb <9 |  |  |  | Level 8 |

|  | Recormon ®  (Epoetin beta,  2000 IU) | Nesp ® (Darbepoietin-alpha,  20 mcg) |
| --- | --- | --- |
| Level 0 | nil | nil |
| Level 1 | QM | nil |
| Level 2 | QOW | nil |
| Level 3 | QW | nil |
| Level 4 | BIW | nil |
| Level 5 | TIW | nil |
| Level 6 | QW | QW |
| Level 7 | nil | BIW |
| Level 8 | QW | BIW |

(Examples: Level 1 prescribes Recormon ® 2000 IU QM; Level 8 prescribes Recormon ® 2000 IU QW and Nesp ® 20 mcg BIW.)

Supplement 2. Iron supplements prescription algorithms in the computerized decision support system (CDSS).

| Hb | ferritin | Iron Saturation  (%) | previous Fe-Back ® | Change in ferritin (σferritin) | Iron (Fe-Back ®) prescription adjustments |
| --- | --- | --- | --- | --- | --- |
| ≥ 12 |  |  |  |  | Level 0 |
|  | Ferritin ≥ 600 |  |  |  | Level 0 |
|  |  | ≥ 50 |  |  | Level 0 |
| 12> Hb ≥ 10 | 600 > Ferritin ≥ 400 |  | ≠ Level 0 | σferritin ≥ 100 | -2 Levels than last prescription |
|  |  |  |  | 100 > σferritin ≥ 0 | -1 Levels than last prescriptionc |
|  |  |  |  | σferritin <0 | No change from last prescription |
|  |  |  | Level 0 |  | Level 1 |
|  | 400 > Ferritin ≥ 200 |  | ≠ Level 0 | σferritin ≥ 0 | +1 Level than last prescription |
|  |  |  |  | σferritin <0 | +2 Levels than last prescription |
|  |  |  | Level 0 |  | level2 |
|  | Ferritin < 200 |  | yes |  | +2 Levels than last prescription |
|  |  |  | Level 0 |  | Level 3 |
| 10>Hb>=9 | 600 > Ferritin ≥ 400 | >=20 | ≠level 0 | σferritin ≥ 50 | -1 Levels than last prescription |
|  |  |  |  | σferritin <50 | No change from last prescription |
|  |  |  | Level 0 |  | Level 1 |
|  |  | <20 | ≠level0 | σferritin ≥ 100 | -1 Level than last prescription |
|  |  |  |  | σferritin <100 | No change from last prescription |
|  |  |  | Level 0 |  | Level 2 |
|  | 400 > Ferritin ≥ 300 | >=20 | ≠ Level 0 | σferritin ≥ 100 | -1 Level than last prescription |
|  |  |  |  | σferritin <100 | No change from last prescription |
|  |  |  | Level 0 |  | Level 2 |
|  |  | <20 | ≠ Level 0 | σferritin ≥ 0 | No change from last prescription |
|  |  |  |  | σferritin <0 | +1 Level than last prescription |
|  |  |  | Level 0 |  | Level 3 |
|  | 300 > Ferritin ≥ 200 | >=20 | ≠ Level 0 | σferritin ≥ 0 | No change from last prescription |
|  |  |  |  | σferritin <0 | +1 Level than last prescription |
|  |  |  | Level 0 |  | Level 3 |
|  |  | <20 | ≠ Level 0 | σferritin ≥ 0 | +1 level than last prescription |
|  |  |  |  | σferritin <0 | +2 Levels than last prescription |
|  |  |  | Level 0 |  | Level 4 |
|  | 200 > Ferritin ≥ 100 | >=20 | ≠ Level 0 | σferritin ≥ 0 | +1 Level than last prescription |
|  |  |  |  | σferritin <0 | +2 Levels than last prescription |
|  |  |  | Level 0 |  | Level 4 |
|  |  | <20 | ≠ Level 0 |  | +3 Levels than last prescription |
|  |  |  | Level 0 |  | Level 5 |
|  | Ferritin <100 |  | ≠ Level 0 |  | +3 Levels than last prescription |
|  |  |  | Level 0 |  | Level 5 |
| 9>Hb | 600 > Ferritin ≥ 500 | >=30 | ≠ Level 0 | σferritin ≥ 50 | -1 Level than last prescription |
|  |  |  |  | σferritin <50 | No change from last prescription |
|  |  |  | Level 0 |  | Level 1 |
|  |  | <30 | ≠ Level 0 | σferritin ≥ 100 | -1 Level than last prescription |
|  |  |  |  | σferritin <100 | No change from last prescription |
|  |  |  | Level 0 |  | Level 2 |
|  | 500 > Ferritin ≥ 400 | >30 | ≠ Level 0 | σferritin ≥ 100 | -1 Level than last prescription |
|  |  |  |  | σferritin <100 | No change from last prescription |
|  |  |  | Level 0 |  | Level 2 |
|  |  | <30 | ≠ Level 0 | σferritin ≥ 0 | No change from last prescription |
|  |  |  |  | σferritin <0 | +1 Level than last prescription |
|  |  |  | Level 0 |  | Level 3 |
|  | 400 > Ferritin ≥ 300 | >=30 | ≠ Level 0 | σferritin ≥ 0 | No change from last prescription |
|  |  |  |  | σferritin <0 | +1 Level than last prescription |
|  |  |  | Level 0 |  | Level 3 |
|  |  | <30 | ≠ Level 0 | σferritin ≥ 0 | +1 Level than last prescription |
|  |  |  |  | σferritin <0 | +2 Levels than last prescription |
|  |  |  | Level 0 |  | Level 4 |
|  | 300 > Ferritin ≥ 200 | >=30 | ≠ Level 0 | σferritin ≥ 0 | +1 Level than last prescription |
|  |  |  |  | σferritin <0 | +2 Levels than last prescription |
|  |  |  | Level 0 |  | Level 4 |
|  |  | <30 | ≠ Level 0 |  | +3 Levels than last prescription |
|  |  |  | Level 0 |  | Level 5 |
|  | Ferritin <200 |  | ≠ Level 0 |  | +3 Levels than last prescription |
|  |  |  | Level 0 |  | Level 5 |

|  | Fe-Back ® (ferric hydroxide sucrose solution, 2%, 5ml, total 100mg) |
| --- | --- |
| Level 0 | No prescription |
| Level 1 | QM |
| Level 2 | QM |
| Level 3 | QOW |
| Level 4 | QW |
| Level 5 | QW |
| Level 6 | QW |
